# High expression of FER tyrosine kinase predicts poor prognosis in clear cell renal cell carcinoma

**DOI:** 10.3892/ol.2012.1032

**Published:** 2012-11-16

**Authors:** CAN WEI, SONG WU, XIANXIN LI, YADONG WANG, RUI REN, YONGQING LAI, JIONGXIAN YE

**Affiliations:** 1 Medical Department, The University of Hong Kong-Shenzhen Hospital;; 2 Department of Urology, Peking University Shenzhen Hospital;; 3 Guangdong and Shenzhen Key Laboratory of Male Reproductive Medicine and Genetics, Institute of Urology, Peking University Shenzhen Hospital, Shenzhen PKU-HKUST Medical Center, Shenzhen;; 4 Medical Department, The First Clinical College, Anhui Medical University, Hefei, P.R. China

**Keywords:** clear cell renal cell carcinoma, FER, tumorigenesis, metastasis, prognosis

## Abstract

FER tyrosine kinase (FER) has been demonstrated to play a critical role in tumorigenesis and metastasis; however, its potential value as a novel prognostic marker for clear cell renal cell carcinoma (ccRCC) remains unclear. In 48 paired samples of ccRCCs and normal adjacent tissues (ADTs), real-time PCR was used to evaluate the expression of FER mRNA. The expression of FER protein was assessed in 87 ADTs and 206 samples of ccRCC using immunohistochemical methods. Statistical analysis was used to examine the correlations between the expression levels of FER and the clinical characteristics of ccRCC patients. A significant difference was identified between ccRCC tissues and ADTs in the mRNA levels of FER. Immunohistochemistry analyses revealed higher expression of FER protein in 87 ccRCC samples compared to the paired ADTs. In addition, FER protein expression in 206 ccRCC samples was significantly correlated with tumor size, T stage, N classification, metastasis, recurrence and Fuhrman grade, while associations with age and gender were not identifed. The Kaplan-Meier survival analysis showed that patients with high FER levels had a poorer survival outcome compared with those with lower levels. The log-rank test demonstrated that the cumulative survival rates were significantly different between the two groups. The Cox regression analysis indicated that FER expression, N stage and distant metastasis were independent prognostic factors for overall survival of ccRCC patients. Our results indicate that overexpression of FER in tumor tissues predicts a poor prognosis of patients with ccRCC, and FER may serve as a novel prognostic marker for ccRCC.

## Introduction

Renal cell carcinoma is the second leading cause of mortality among urinary tumors, accounting for 2% of adult malignancies, of which the most common subtype is clear cell renal cell carcinoma (ccRCC) (1). ccRCC affects approximately 150,000 individuals each year and causes approximately 78,000 mortalities globally with increasing incidence and mortality. More than one-third of patients may have metastasis when diagnosed, and 50% of patients may suffer recurrence even after nephrectomy (2). In recent years, specific prognostic markers for ccRCC, including Ki-67, MCM2, SAT1, L1CAM and BIRC5, have emerged, but large-scale clinical application is impossible. Despite the continual progress in medical technology, the clinical characteristics of ccRCC remain difficult to predict (3). Therefore, novel diagnostic and prognostic markers of ccRCC could be valuable in high-risk individuals and those with existing disease.

Protein tyrosine phosphorylation plays a important role in signal transduction. This process contains numerous complicated processes. A recent study revealed the corelations between abnormal protein tyrosine phosphorylation and tumorigenesis, invasion and metastasis (4). Therefore, studies on tyrosine kinases could be significant for acquiring insights into the underlying mechanisms of oncogenesis, distant metastasis and recurrence.

FER, a 94-kDa protein, belongs to the subfamily of non-receptor protein tyrosine kinases. Similar to other tyrosine kinases, FER has a central Src-homology 2 (SH2) domain involved in binding to phosphotyrosine-containing peptide sequences and a highly conserved C-terminal kinase domain. It is distinguished from other tyrosine kinases by an NH_2_-terminal FER/ClP4 homology and adjacent coiled-coil domains (5,6). FER exists ubiquitously in human cells and participates in the signaling processes of cell proliferation, differentiation, apoptosis, movement and adhesion (5). However, a number of studies have identified higher levels of FER in cancer cells compared with adjacent normal cells (7,8); the underlying mechanisms of which have remained unclear to date. Moreover, the correlation between FER expression and prognosis status remains ambiguous. In the present study, we examine the expression and clinical significance of FER in ccRCC and explore the association between FER expression level and prognosis status.

## Materials and methods

### Patients and tissue samples

The study was approved by the institutional review board and ethical committee of Peking University Shenzhen Hospital, Shenzhen, China. All patients participated after providing written informed consent. For real-time qPCR, 48 paired samples of ccRCCs and normal adjacent tissues (ADTs) were collected from patients who underwent radical nephrectomy at the Department of Urology, Peking University Shenzhen Hospital between November 2009 and December 2011. The ADTs were located 2.0 cm away from visible ccRCC lesions. The fresh tissue samples were immediately immersed in RNAlater (Qiagen, Hilden, Germany) following surgical resection and stored at 4°C overnight and then frozen in liquid nitrogen and stored at −80°C until analysis.

For immunohistochemical analysis of FER protein, a total of 206 paraffin-embedded samples of pathologically verified ccRCC and 87 adjacent normal renal tissue samples were collected. All patients received radical nephrectomy at the Department of Urology, Sun Yat-Sen University Cancer Center, Guangzhou, China between June 2000 and September 2010. The histological and clinical diagnoses of the tumors in all patients were performed by the Pathology Department of Sun Yat-Sen University Cancer Center. The characteristics of the 206 patients are listed in Table I. The survival information of the 206 patients was collected over the telephone. The clinical characteristics of the patients were obtained from patient medical records. Tumor stage was reclassified based on the 2011 Union for International Cancer Control (UICC) TNM classification of malignant tumors, and nuclear grading was performed according to Fuhrman’s system (9).

### Real-time qPCR

Total RNA was extracted from 48 paired ccRCC samples and normal tissue by TRIzol (Invitrogen Life Technologies, Carlsbad, CA, USA) according the manufacturer’s instructions. Then Omniscript RT kit (Qiagen) was used to synthesize the first-strand cDNA. The total reaction volume was 20 *μ*l including 1 *μ*g RNA, and the reaction mixture was incubated at 42°C for 60 min, heated at 95°C for 10 min and then cooled on ice. The RNA and cDNA were evaluated using an Agilent 2100 Bioanalyzer (Agilent Technologies, Santa Clara, CA, USA). The corresponding primer sequences were as follows: FER sense primer, 5′-TTCGAGGGCACTGGGTTTTC-3′; reverse primer, 5′-TTCCCTTGCCCAGTAATTCTCC-3′. GAPDH sense primer, 5′-GGAGTCCACTGGCGTCTTCACC-3′; reverse primer, 5′-GAGGAGTGGGTGTCGCTGTTG-3′. Real-time PCR was conducted using SYBR Green dye in a 7000 Sequence Detection System (Applied Biosystems, Carlsbad, CA, USA). The 20-*μ*l real-time PCR mixture contained 1 *μ*l of cDNA (synthesized as described above), 10 *μ*l SYBR Green Master mix (Invitrogen) and 1 *μ*l of each upstream and downstream primer. The amplification conditions were 95°C (2 min) for 1 cycle and 95°C (5 sec), 57°C (30 sec) and 68°C (30 sec) for 40 cycles. Relative expression levels of FER were normalized to the geometric mean of GAPDH (internal control gene). The data were analysed via the comparative threshold cycle (2^−ΔCT^, − ΔCT=CT*_FER_*−CT*_GAPDH_*) method (10).

### Immunohistochemical analysis

Immunohistochemistry was performed to examine FER expression in the 206 ccRCC samples and 87 paired samples of ADTs. All procedures were performed using standard techniques. Briefly, paraffin-embedded specimens were cut into 5-*μ*m sections and heated at 65°C for 30 min. The sections were deparaffnized in xylene and rehydrated in a descending ethanol series. Endogenous peroxidase activity was blocked with 3% hydrogen peroxide for 20 min. The sections were boiled in 10 mmol/l citrate buffer (pH 6.0) to unmask the epitopes.Non-specific protein binding was performed by incubations with 10% bovine serum albumin for 30 min. For the detection of FER, the sections were incubated with the polyclonal rabbit anti-human FER antibody (Abcam, Cambridge, MA, USA) diluted at 1:400, and then incubated overnight at 4°C. The negative control was performed by replacing the primary antibody with antibody diluent. They were then rinsed with PBS and incubated using a anti-Rabbit Immunohistochemistry kit (Maixin Bio., Fujian, China) at 37°C for 20 min. After rinsing with PBS, the tissue sections were stained for 5 min with 3,3′-diaminobenzidine tetrahydrochloride (DAB), counterstained with hematoxylin, dehydrated and then mounted in Crystal Mount (Maixin Technologies, Fuzhou, China).

### Staining evaluation

The stained sections were reviewed by two independent observers who had no prior knowledge of the clinicopathological data of the patients. The scoring was based mainly on color intensity and extensity. The proportion of cells expressing FER varied from 0 to 100%, and the intensity of staining varied from weak to strong. The proportion of FER expression tumour cells was scored at low magnification on a scale of 0–5 (0, no cells positive; 1, 0–5% of cells positive; 2, 6–25%; 3, 26–50%; 4, 51–75%; 5, 76–100%). The intensity score was determined at high magnification on a scale of 0–3 (0, negative staining; 1, weakly positive staining; 2, moderately positive staining; 3, strongly positive staining), and the final score was calculated by the multiplication of the two parameters, with scores of 0, 1, 2, 3, 4, 5, 6, 8, 9, 10, 12 and 15. We set the optimal cut-off values for FER level by measuring heterogeneity in overall survival rates via the log-rank test method, and designated low expression as total score <5, high expression as total score ≥5. Thus, the stained sections were divided into two different groups.

### Statistical analysis

Paired-sample t-tests were used in the RT-PCR and immunohistochemistry assays to analyze the significance of the differences in mRNA level and protein expression between ccRCCs and the adjacent normal tissues. The correlation between FER expression and clinical and pathological characteristics was assessed using the χ^2^ test. Survival curves were plotted according to the Kaplan-Meier method and compared by the log-rank test. Multivariate analysis according to Cox’s proportional hazards regression model adjusted for clinicopathological factors (age, gender, tumor size, Fuhrman grade, TNM stage and FER expression) was performed to assess which clinical variables were independently correlated with overall survival. Statistical analysis was performed using the SPSS 17.0 package. P<0.05 was considered to indicate a statistically significant difference.

## Results

### RT-PCR analysis of FER mRNA in 48 ccRCC tumor samples

**Figure 1.**
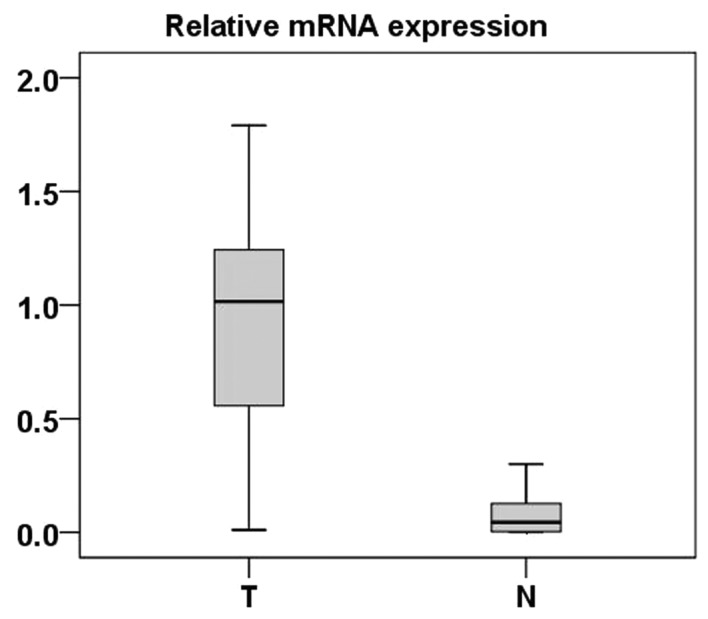
Real-time quantitative RT-PCR analysis of FER expression. The relative expression of FER mRNA in RCC tumor (T) tissue samples was higher than that in the paired ADTs (N) (n=48; P<0.001). The bottom and the top of the box represent the 25th and the 75th percentile, respectively, and the band near the middle of the box is the 50th percentile (the median). The ends of the whiskers represent the 2.5th percentile and the 97.5th percentile.

Real-time PCR was performed to measure the expression of FER mRNA in 48 ccRCC tumor tissues and ADTs. Compared with normal tissues, 46 ccRCC tumor tissues exhibited significantly high expression of FER at the mRNA level (P<0.001, paired-sample t-test) (Fig. 1).

### Immunohistochemical analysis of FER expression in 87 ccRCC samples and the paired ADTs

**Figure 2.**
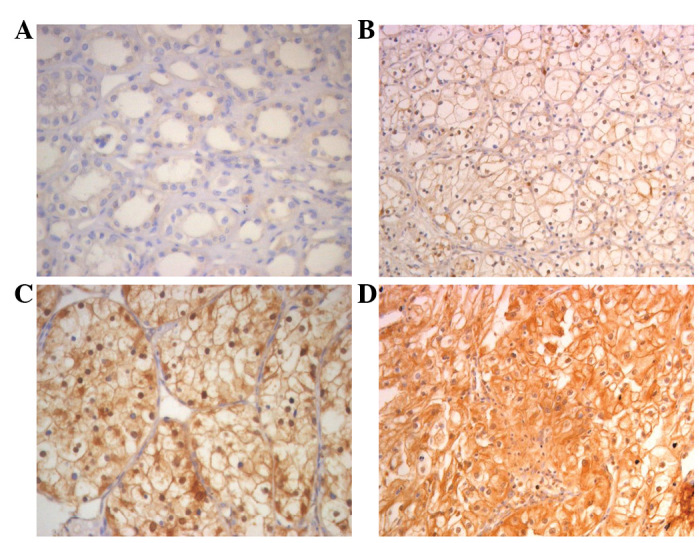
Immunohistochemical analysis of the expression of FER protein. FER is mainly localized within the nuclei and cytoplasm. (A) Immunostaining of the ADT samples was negative or at a very low level. (B) Weak FER staining in cancerous tissue. (C) Moderate FER staining in cancerous tissue. (D) Strong FER staining in most of the tumor cells. Magnification, ×400. ADT, normal adjacent tissues.

**Figure 3.**
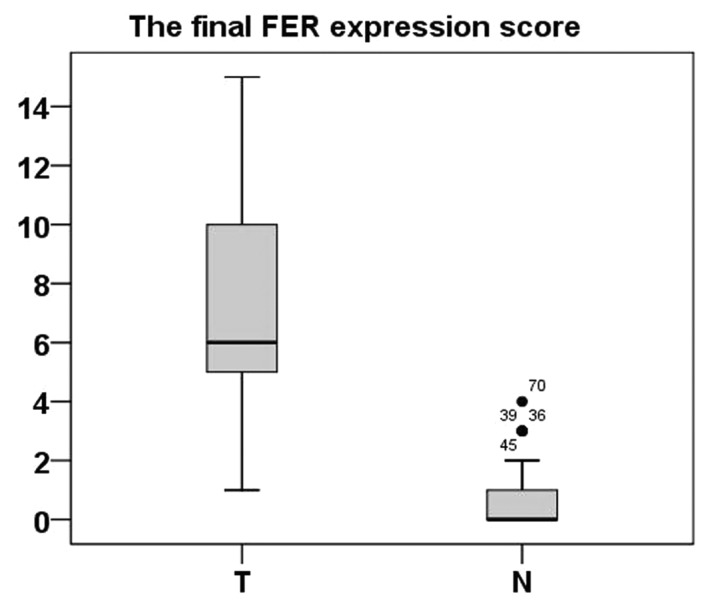
Increased protein expression of FER in ccRCC. The relative protein expression of FER in ccRCC tumor (T) tissue samples was higher than that in the paired ADT samples (N) (n=87, P<0.001). The bottom and top of the box are the lower and upper quartiles, and the band near the middle of the box is the median. The ends of the whiskers represent the 2.5th percentile and the 97.5th percentile. Four black spots represent the special value outliers. ccRCC, clear cell renal cell carcinoma; ADT, normal adjacent tissues.

Immunohistochemistry was applied to assess the expression and subcellular localization of FER protein in 87 paraffin-embedded ccRCC tissues and 87 paired ADTs. As shown in Fig. 2, FER staining was present mainly in the nuclei and cytoplasm (Fig. 2). In the normal renal tissues, FER expression was negative (52/87; score, 0) or at a low level (35/87; score, ≤5). FER expression in the 83 tumor tissue samples was higher than the ADTs (P<0.001, paired-sample t-test) (Fig. 3).

### Immunohistochemical analysis of the correlation between FER protein expression and clinical features in 206 ccRCC tumor samples

Immunohistochemical analysis was performed in 206 paraffin-embedded ccRCC tumor samples to further assess the correlation between FER expression and various clinicopathological parameters. As shown in Table I, low expression of FER (score, ≤4) was demonstrated in 70 of the 206 tumor samples, while high expression (score, ≥5) was demonstrated in a further 136 samples. Increased expression of FER in tumor samples was correlated with tumor size (χ^2^=8.161; P=0.004), T stage (χ^2^=8.542; P=0.014), N classification (χ^2^=6.131; P=0.013), metastasis (χ^2^=6.680; P=0.010), recurrence (χ^2^=8.959; P=0.003) and Fuhrman grade (χ^2^=12.374; P=0.006), while associations with age (χ^2^=1.227; P=0.268) and gender (χ^2^=0.168; P=0.682) were not identified. High expression of FER was revealed in 45.8, 58.8 and 87.9% of T1, T2, and T3/4 stage ccRCCs, respectively (P=0.014; χ^2^ test). High expression of FER was observed in 58.2 and 77.4% of ccRCCs with size <7 cm and ≥7 cm, respectively (P=0.004; χ^2^ test). High expression of FER was observed in 62.7 and 86.2% of N0 and N1/2 stage ccRCCs, respectively (P=0.013, χ^2^ test). High expression of FER was observed in 88.5 and 62.8% of ccRCCs with or without metastasis respectively (P=0.010, χ^2^ test). High expression of FER protein was observed in 88.2 and 61.6% of ccRCCs with or without recurrence, respectively (P=0.003; χ^2^ test).

### Survival analysis

**Figure 4.**
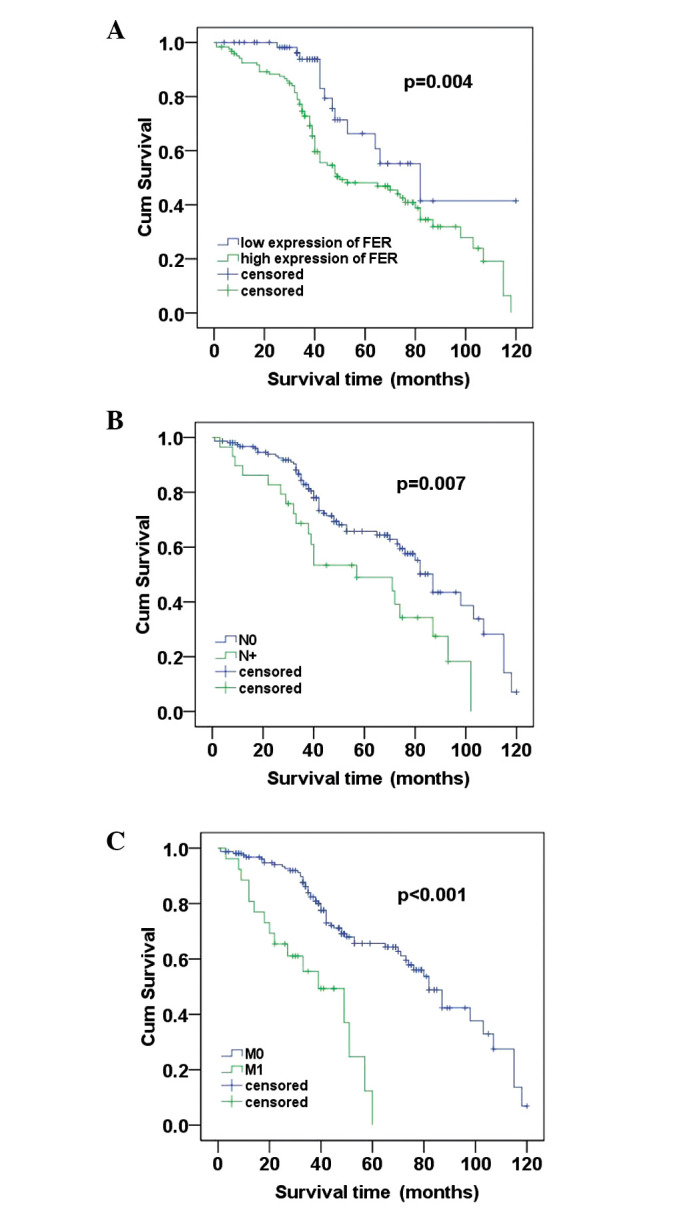
(A) Survival analysis of primary ccRCC patients (n=206). Kaplan-Meier survival analysis of primary ccRCC patients after surgical resection with high FER expression (n=136) and low expression (n=70). The cumulative survival rate for patients in the FER high group was significantly lower than that for patients in the FER-negative group (χ^2^=8.315; P=0.004). (B) Patients with no regional lymph node involvement (N0) had a better prognosis than those with regional lymph node involvement (N+) (χ^2^=7.251; P=0.007). (C) Patients with no distant metastasis (M0) had a high cumulative survival rates when compared with patients with metastasis (M1) (χ^2^=14.214; P<0.001). The longest follow-up time was 120 months. ccRCC, clear cell renal cell carcinoma.

To further investigate the prognostic value of FER expression in ccRCC, Kaplan-Meier analysis and the log-rank test were applyed to assess the correlation between FER expression level in ccRCC and prognosis status. We identified that the level of FER expression was correlated with the overall survival of ccRCC patients. Individuals with a higher level of FER expression had poorer survival rates compared with those with a lower level. The mean survival time in the group of highly expressed FER patients was 64.840 months and the median survival time was 50 months, but the mean and median survival time in the low expression group time were 90.331 and 89 months, respectively. The results of the log-rank test demonstrated that the survival rates were significantly different between these two groups (log-rank, P=0.004) (Fig. 4A). Furthermore, patients with no regional lymph node involvement (N0) had a better prognosis than those with regional lymph node involvement (N+; log-rank, P=0.007; (Fig. 4B). Similarly, patients with no metastasis (M0) had a high cumulative survival rates compared with patients with metastasis (M1; log-rank, P<0.001) (Fig. 4C).

In addition, the multivariate Cox regression analysis indicated that FER expression (P=0.028), N stage (P=0.009) and distant metastasis (P<0.001) were independent prognosis factors for overall survival of ccRCC patients (Table II).

## Discussion

ccRCC is the most common subtype of renal tumor, accounting for 70–80% of all RCCs. Incidence of ccRCC has increased markedly in the last two decades, and the annual mortality has become significantly higher than other tumors in the genitourinary tract (11).

The clinical outcome of ccRCC remains poor despite advances in clinical technologies (12). The TNM staging system and other clinicopathological parameters have limited value in predicting the prognosis of individuals with ccRCC, and approximately 50% of those patients with less advanced disease are thought to experience metastasis even after nephrectomy (13). Therefore, it is extremely significant to identify specific molecular biomarkers of ccRCC for early diagnosis and evaluation of prognosis.

FER is a member of the non-receptor protein tyrosine kinases subfamily. FER is expressed ubiquitously in a variety of tissues and cells and is overexpressed at a markedly high level in malignant tumors (5,7), including prostate cancer and hepatocarcinoma (7,8). Overexpression of *Drosophila* FER may induce rodent fibroblasts canceration (14). However, since its discovery in 1988 (15), studies on the functional regulation mechanism of FER in malignancy have been relatively limited, and the focus has been mainly set on cell adhesion. The part FER plays in the regulation of integrin-mediated focal adhesion and cadherin-mediated intercellular adhesion has been confirmed. FER may promote CTNNB1 dephosphorylation and E-cadherin-mediated adhesion stability under normal circumstances. However, high expression of FER may directly induce CTNNB1 phosphorylation, resulting in a disintegration of cadherin-mediated adhesion (16,17). FER may integrate with the integrin and cadherin complex via phosphorylation of cortactin, a crucial molecule in tumor cell metastasis (18,19). Notably, FER dissociated from the cadherin complex could be recruited to the integrin complex, leading to p130CAS dephosphorylation and blocking of integrin-mediated adhesion (17) .

In addition, overexpressed FER could phosphorylate EGF receptor and activate the EGF-mediated NF-κB signaling pathway, which is crucial for cancer cell survival and proliferation (20). Downregulation of FER via antisense cDNA impairs prostate cancer cells growth and colony formation *in vitro*(21). RNA interference against FER may also arrest the mitotic cycle in G0–G1 phase*in vitro*(22).

Thus a high level of FER may disintegrate integrin/cadherin-mediated intercellular adhesion and promote metastasis. In accordance with this, our results demonstrate a high distant metastasis and recurrence rate in ccRCC patients with a high FER level, which suggests a greater metastasis and invasion ability in tumor cells with overexpressed FER.

To the best of our knowledge, this is the first study to indicate the clinical significance of FER in ccRCC. Real-time PCR in 48 ccRCC tumour tissues and paired ADT samples revealed a significant increase in FER mRNA in ccRCC samples. Further immunohistochemical analysis in 87 paired samples of ccRCCs and ADTs confirmed overexpression of FER protein in tumor tissue. These results indicate that FER may play important roles in the initiation and progression of malignancies.

To further investigate the prognostic value of FER, immunohistochemical analysis was performed to evaluate the correlation between FER expression and various clinicopathological parameters. In the present study, we demonstrated that an increased level of FER expression was significantly correlated with tumor size, Fuhrman grade, stage, N classification, metastasis and recurrence. According to the results of Kaplan-Meier analysis, the FER protein expression level in ccRCC was significantly correlated with overall survival. Patients with a high FER expression level had a shorter survival time than those with a low FER level. The log-rank test revealed that the group with a lower expression of FER had a more favorable prognosis than the higher expression group. The TNM stage of ccRCC was closely correlated with prognosis (23). Consistent with this, in this study, FER expression, N classification and distant metastasis were independent prognosis factors for overall survival of ccRCC patients by multivariate Cox regression analysis. Therefore, this study reveals that there are significant correlations between FER expression level and clinicopathological parameters and may be a potential prognostic marker and therapeutic target for ccRCC.

Fuhrman’s nuclear grading system is considered to be a reliable prognostic indicator for ccRCC (9). However, the multivariate Cox regression analysis in our study did not reveal any correlations between Fuhrman grade and prognosis, this may due to our limited sample size or observation error made by the pathologists.

This is the first study aimed at evaluating the possibility of using FER as a clinically potential indicator for disease progression, as well as a prognostic marker for patient survival in ccRCC. However, it should be acknowledged that this study was a single hospital-based, retrospective study, and therefore, multicenter or community-based prospective studies are required.

In conclusion, our findings indicate that the expression levels of FER in ccRCC tissues are significantly higher than those in ADTs. Moreover, high levels of FER were associated with poor survival in patients with advanced ccRCC. Multivariate survival analysis showed that FER is a potential prognostic marker for ccRCC. FER, therefore, may provide a valuable marker in the prognostic evaluation of ccRCC.

## Figures and Tables

**Table I.** Association between FER and clinicopathological characteristics in ccRCC patients.

		FER			
Parameters	n	High	Low	χ^2^	P-value
Total	206	136	70	0.168	0.682
Gender					
Male	145	97	48		
Female	61	39	22		
Age (years)					
≥50	137	94	43	1.227	0.268
<50	69	42	27		
Size (cm)				8.161	0.004
≥7	84	65	19		
<7	122	71	51		
T stage				8.542	0.014
T1	139	87	52		
T2	34	20	14		
T3/4	33	29	4		
N stage				6.131	0.013
N0	177	111	66		
N+	29	25	4		
Metastasis				6.680	0.010
No (M0)	180	113	67		
Yes (M1)	26	23	3		
Recurrence				8.959	0.003
No	172	106	66		
Yes	34	30	4		
Fuhrman				12.374	0.006
1	37	18	19		
2	124	81	43		
3	25	20	5		
4	20	18	2		

ccRCC, clear cell renal cell carcinoma.

**Table II.** Multivariate Cox regression analysis for the overall survival rates of ccRCC patients.

Risk factors	Relative risk	95% Confidence interval	P-value
T stage	1.291	0.545–1.143	0.266
N stage	1.993	1.185–3.417	0.009
M stage	4.257	2.264–9.863	<0.001
Age	0.621	0.505–1.002	0.137
Size	0.840	0.603–1.578	0.740
Gender	0.714	0.481–1.173	0.306
Fuhrman Grade	9.108	1.398–7.601	0.122
FER expression	0.560	0.437–0.964	0.028

ccRCC, clear cell renal cell carcinoma.
